# Supplementary material for: RAPIDprep: A Simple, Fast Protocol for RNA Metagenomic Sequencing of Clinical Samples
Source: Viruses. 2023 Apr 19;15(4):1006. doi: 10.3390/v15041006 (PMC10146689; doi:10.3390/v15041006)
Supplement: Supplementary file 1 [file viruses-15-01006-s001.zip › viruses-2143229-supplementary.pdf]

Article

RAPID*prep*: A Simple, Fast Protocol for RNA Metagenomic Sequencing of Clinical Samples

Rachel L. Tulloch [1,2,†], Karan Kim [1,2,†], Chisha Sikazwe [3,4], Alice Michie [3,4], Rebecca Burrell [2,5], Edward C. Holmes [2], Dominic E. Dwyer [2,6], Philip N. Britton [2,5], Jen Kok [6] and John-Sebastian Eden [1,2,*]

1 Centre for Virus Research, Westmead Institute for Medical Research, Westmead, NSW 2145, Australia
2 Sydney Institute for Infectious Diseases, School of Medical Sciences, The University of Sydney, Sydney, NSW 2006, Australia
3 PathWest Laboratory Medicine WA, Department of Microbiology, Nedlands, WA 6009, Australia
4 School of Biomedical Sciences, The University of Western Australia, Crawley, WA 6009, Australia
5 Departments of Infectious Diseases and Microbiology, The Children's Hospital at Westmead, Westmead, NSW 2145, Australia
6 NSW Health Pathology Institute for Clinical Pathology and Medical Research, Westmead Hospital, Westmead, NSW 2145, Australia
* Correspondence: js.eden@sydney.edu.au; Tel.: +61-2-8627-1817
† These authors contributed equally to this work.

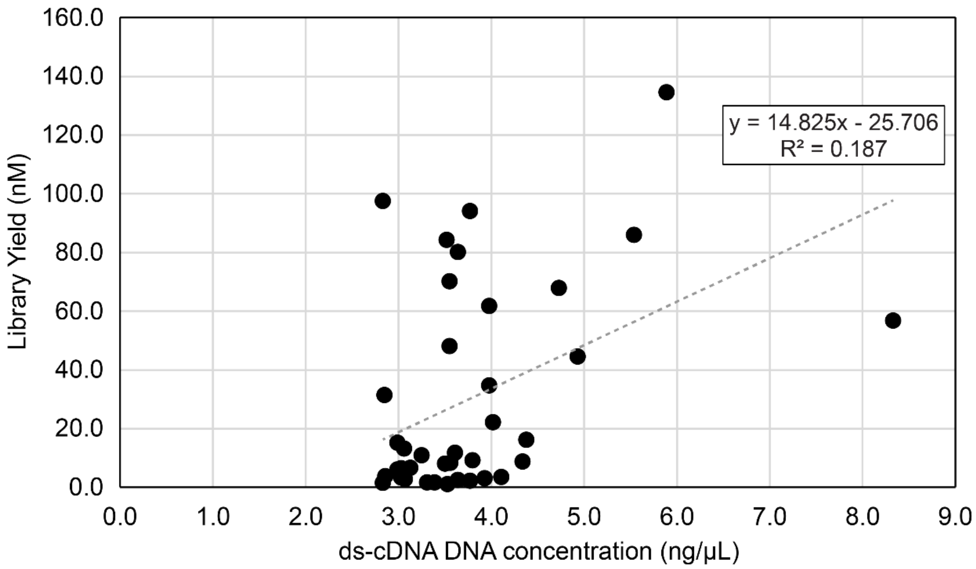

Supplementary Figure S1 – Linear regression showing correlation between input ds-DNA concentration and library yield for the large RAPID*prep* run (n=40 respiratory libraries). The DNA concentration of the rRNA-depleted ds-cDNA libraries (ng/uL) – x-axis – was measured using the Qubit high sensitivity dsDNA assay and compared to the final library yield (nM) – y-axis – measured using the Agilent TapeStation. Linear regression was performed to determine correlation between the two measurements (grey dotted line) with the key showing the slope equation and goodness of fit (R^2).

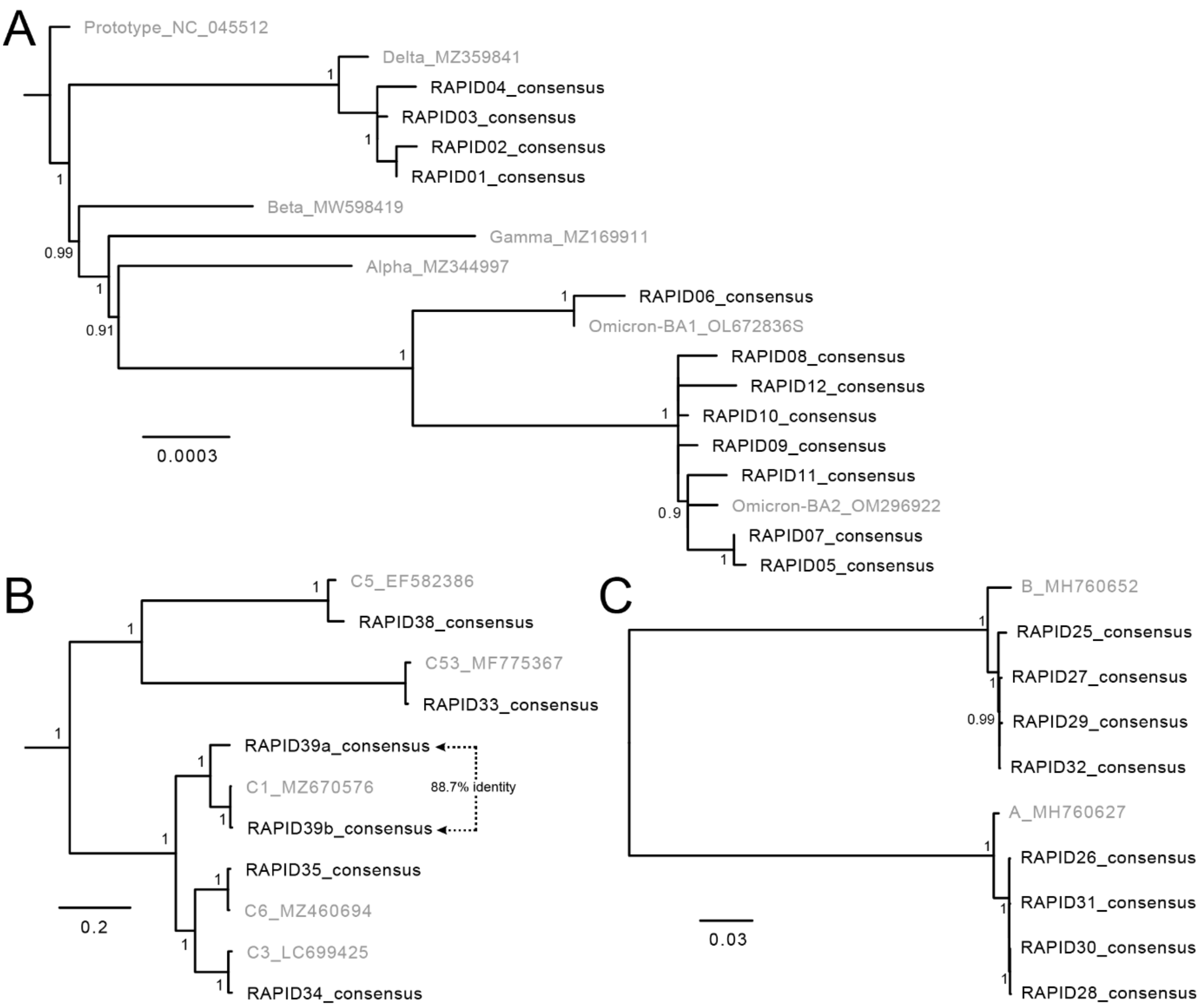

Supplementary Figure S2 – Phylogenetic analysis of viruses identified by RAPID*prep* assay. Maximum likelihood phylogenies were estimated for (**a**) SARS-CoV-2, (**b**) rhinovirus and (**c**) RSV strain identified in this study (black text) against known reference strains (grey text). The GTR + Gamma DNA substitution model was used with 1000 bootstrap replicates to measure support (proportions indicates at each node). Each sequence is labelled according to the library name with multiple viruses indicated with an additional letter (i.e. RAPID39a and RAPID39b). The scales are proportional to the number of substitutions per site.

Supplementary Table S1 – Samples sequenced using RAPIDprep and a commercial assay

**Sample**	**RAPID*prep***		**SMARTer Stranded Total RNA-Seq Kit v2**	
	Library	*NCBI SRA accession*	*Library*	*NCBI SRA accession*
Zymo_2	RAPID15	SRR22726250	ICU15	SRR22838413
Zymo_3a	RAPID16	SRR22726249	ICU16	SRR22838412
ICU-001_1	RAPID17	SRR22726248	ICU17	SRR22838439
ICU-002_1	RAPID18	SRR22726247	ICU18	SRR22838438
ICU-007_1	RAPID19	SRR22726246	ICU19	SRR22838437
ICU-007_2	RAPID20	SRR22726245	ICU20	SRR22838436
ICU-007_3	RAPID21	SRR22726243	ICU21	SRR22838435
ICU-075_1	RAPID22	SRR22726242	ICU22	SRR22838423
ICU-077_1	RAPID23	SRR22726241	ICU23	SRR22838422
ICU-102_1	RAPID24	SRR22726240	ICU24	SRR22838417

Supplementary Table S2 – Genomic yield, coverage, recovery and qPCR results

Library	Virus	Non-host, non-rRNA	Viral reads			Coverage		qPCR
		reads	*reads*	*RPM*	*log(RPM)*	*depth X*	*genome %*	*Ct*
RPD01	SARS-CoV-2	2,834,326	1,792,076	632,275.89	5.80	9,159.00	100.00	22.46
RPD02	SARS-CoV-2	1,461,808	4,552	3,113.95	3.49	21.50	99.50	30.23
RPD03	SARS-CoV-2	4,932,332	15,696	3,182.27	3.50	70.30	99.50	33.92
RPD04	SARS-CoV-2	3,530,136	2,768,186	784,158.46	5.89	15,815.00	100.00	20.37
RPD05	SARS-CoV-2	3,592,638	34,308	9,549.53	3.98	159.50	100.00	31.00
RPD06	SARS-CoV-2	3,170,060	2,651,566	836,440.32	5.92	13,626.00	100.00	23.47
RPD07	SARS-CoV-2	3,517,722	2,336,874	664,314.58	5.82	12,450.00	100.00	22.16
RPD08	SARS-CoV-2	1,723,666	1,470,634	853,201.26	5.93	7,675.00	100.00	22.46
RPD09	SARS-CoV-2	4,502,692	4,334,060	962,548.63	5.98	23,085.00	100.00	20.78
RPD10	SARS-CoV-2	119,716	9,580	80,022.72	4.90	46.30	99.90	30.57
RPD11	SARS-CoV-2	789,160	51,130	64,790.41	4.81	254.30	99.97	29.85
RPD12	SARS-CoV-2	988,654	949,790	960,689.99	5.98	4,877.00	100.00	21.75
RPD25	RSV	403,584	176	436.09	2.64	1.20	41.90	24.15
RPD26	RSV	269,136	2,466	9,162.65	3.96	22.90	99.97	19.20
RPD27	RSV	294,604	518	1,758.29	3.25	4.40	77.80	22.54
RPD28	RSV	316,712	248	783.05	2.89	2.30	72.00	21.23
RPD29	RSV	66,036	804	12,175.18	4.09	7.70	90.10	21.60
RPD30	RSV	5,898	1,042	176,670.06	5.25	10.10	98.50	15.21
RPD31	RSV	17,584	816	46,405.82	4.67	7.90	93.80	17.18
RPD32	RSV	134,974	188	1,392.86	3.14	1.80	54.80	22.38
